# Bioactive Compounds and Their Effect on Blood Pressure—A Review

**DOI:** 10.3390/nu12061659

**Published:** 2020-06-03

**Authors:** Bartosz Malinowski, Raul Ignacio Fajardo Leighton, Christopher George Hill, Paweł Szandorowski, Michał Wiciński

**Affiliations:** Department of Pharmacology and Therapeutics, Faculty of Medicine, Collegium Medicum in Bydgoszcz, Nicolaus Copernicus University, M. Curie 9, 85–090 Bydgoszcz, Poland; fajardoleighton@gmail.com (R.I.F.L.); cghillkittelsen@gmail.com (C.G.H.); pa.szandorowski@gmail.com (P.S.); wicinski4@wp.pl (M.W.)

**Keywords:** nutrition, hypertension, blood pressure, therapy

## Abstract

Elevated blood pressure affects a great part of the elderly population and is the leading risk factor for cardiovascular disease. New approaches have been taken in the fight against this growing problem, in the form of diets (Mediterranean, Dietary Approaches to Stop Hypertension (DASH) and intermittent fasting). Recent research has shown the promising results regarding diets and their effect on the prevention and improvement of elevated blood pressure. This review attempts to take this a step further, reviewing 26 studies in the search for dietary elements that may be causing this improvement. Although good evidence was found in favor of lycopene, Docosahexaenoic acid (DHA), fiber and anthocyanin, further evidence is needed before any conclusions can be made. In contrast, the evidence shows that licorice increases blood pressure.

## 1. Introduction

Elevated blood pressure, also known as, hypertension is a medical condition affecting over 1.13 billion people worldwide and causes approximately 9.4 million deaths due to complications every year [1,2].

This condition may affect people of all ages but has a predominant prevalence in the adult population, affecting over 50% of adults between 50–59 years of age and approximately 70% of adults over 70 years of age [3]. In the older population, hypertension affects almost equally men and women, although there is a slight predominance in men over women (one in four and one in five, respectively) [2]. Recent studies have classified high systolic blood pressure (SBP) as the leading risk factors for death and disability worldwide, as well as hypertension being the largest risk factor for cardiovascular disease [4,5].

Hypertension is defined in office as blood pressure (BP) of > 140 mmHg systolic and/or with >90 mmHg diastolic, or an average blood pressure of > 130/80 mmHg based on 24 h ambulatory blood pressure monitoring (ABPM) [6].

Hypertension can be classified based on etiology. Primary or essential hypertension is the most common type and is responsible for over 90% of cases, but this type of hypertension has a rather unknown heterogenic origin, although, certain studies have linked genetically, environmental and life factors as possible causes for this disease [7,8]. Secondary hypertension accounts for 5–10% of all hypertensive patients and has an identifiable origin which can be treated and even curable if the underlying cause is treated (examples are primary aldosteronism, pheochromocytoma, renal artery stenosis, polycystic ovary syndrome, obstructive sleep apnea and oral contraceptives) [9].

Current European guidelines for the treatment of hypertension advises all hypertensive patients disregarding of their blood pressure to adopt a change in their life-style, which includes the following: a decrease in sodium consumption (< 5 g/days), moderate alcohol consumption, weight reduction, regular physical activity, smoke cessation and other dietary changes, the latest being the object of this review [5]. Current pharmacological treatments have undoubtedly shown great improvements in patients with hypertension, yet, it has been estimated that approximately 32.5% of these patients could achieve a controlled blood pressure, meaning a blood pressure < 140/90 mmHg [10]. With this information in hand, it can be assumed that there is significant room for improvement in the treatment of hypertension. 

Dietary changes are important in the fight against hypertension and diets have been a way of implementing these changes. It is well known that the Mediterranean diet has several cardio protective benefits, such as decreasing incidence of cardiovascular disease, decreasing systolic and diastolic blood pressure (DBP), decreasing all causes of death and decreasing risk of stroke [11]. Intermittent fasting is another diet that has gained popularity and it has been connected with a decrease in total cholesterol, triglycerides, low-density lipoprotein (LDL) cholesterol and a decrease in blood pressure, as well as an inverse correlation with atherosclerosis [12]. The dietary approaches to stop hypertension (DASH) are other diets that have shown significant results in lowering blood pressure [13]. With this information in hand, further research regarding single compounds is necessary in order to improve the current dietary recommendations. In this review, we explore the evidence available of different bioactive compounds that are largely available in our diet, as well as popular in our society regarding their potential benefit in the combat against hypertension.

## 2. Materials and Methods

### 2.1. Data Sources and Searches

All independent authors searched PubMed and MEDLINE for all published results between January 1990 and December 2019 on bioactive compounds and their effect on blood pressure. The following search terms were used: “blood pressure diet” (MeSH Terms) OR “blood pressure” (All Fields) AND “diet” (All Fields) AND “anthocyanian” (All Fields) OR “blood pressure anthocyanian” (All Fields) AND “lycopene” (All Fields) AND “blood pressure lycopene” (All Fields) AND “docosahexaenoic acid” (All Fields) OR “blood pressure DHA” (All Fields) AND “caffeine” (All Fields) OR “blood pressure caffeine”(All fields) AND “dietary fiber”(All fields) OR “blood pressure dietary fiber”(All fields) AND “licorice”(All fields) OR “blood pressure licorice” (All fields). Investigators also searched the bibliographies of relevant articles.

### 2.2. Eligibility Criteria

The present review was prepared accordingly to the following criteria:

- Population: individuals with hypertension, normotension;
- Intervention: individuals with supplementation of described nutrients;
- Comparison: individuals without supplementation of any nutrients;
- Outcome: presence/absence of changes in blood pressure;

All published studies were only included if they were written in English. Included papers could additionally be studies performed on animals. 

### 2.3. Study Selection

Investigators independently reviewed each study’s title and abstract according to the prespecified eligibility criteria. Abstracts of interest were included for full-text analysis. Afterwards, authors analyzed all full-text articles and rejected those that did not meet the aforementioned criteria. Any inconsistencies between the reviewers were resolved by discussion with a supervisor (M.W.).

### 2.4. Data Collection Process and Data Items

All included articles were analyzed independently by the authors. The abstracted information included author names, year of publication, study design, intervention, dose of intervention and main outcomes. The supervisor (M.W.) checked the abstracted information and resolved any disagreements.

## 3. Results

### 3.1. Anthocyanin

Anthocyanins are phytochemicals belonging to the polyphenol family. They are naturally occurring water-soluble pigments ranging from red-orange to purple and are found in different fruits and plants, the most common being cyanidin, pelargonidin, peonidin and delphinidin [14]. There is substantial evidence showing the antioxidant and anti-inflammatory qualities of these polyphenols, thus proving their potentially beneficial health effects [15,16,17]. Although there are few studies regarding the beneficial cardio-protective qualities of anthocyanins, some have reported decreased vascular relaxation of aortic rings and mesenteric vascular beds in rats, reduced risk of myocardial infarction (MI) in young and middle-aged women and lower risk of cardiovascular disease (CVD) in men [18,19,20].

Together with the above-mentioned qualities, in vitro studies suggest potential vasodilating qualities through the enhancement of endothelial nitric oxide synthase and nitric oxide production [21,22]. Endothelial nitric oxide synthase (eNOS) is an enzyme found in vascular endothelial cells and plays a major role in the production of nitric oxide (NO), which causes vasorelaxation of adjacent smooth muscle cells. Evidence suggests that the disruption of eNOS through DNA damage and oxidative stress may be involved in endothelial dysfunction and subsequent hypertension progression [23,24,25].

In a large prospective study from 2011 done by Cassidy et al., a selected population of 157,957 participants from across three cohorts, Nurses Health Study I (NHS I), Nurses Health Study II (NHS II) and from the Health Professionals Follow-up Study (HPFS) were assessed in order to find a relation between flavonoid intake and hypertension incidence. During a 14-year period, the participants answered a follow-up dietary intake questionnaire in order to assess their daily flavonoid intake. The mean flavonoid intake across cohorts was 358–413 mg/day from which 12.5–15.2 mg/day were anthocyanins. The participants were separated based on their total flavonoid intake, which was measured with the US Department of Agriculture (USDA) database. The final observations of this research reported an association between increased consumption of anthocyanins and a reduced risk of hypertension development in the highest quintile of 8% compared to the lower quintile *p* < 0.03 [26].

In 2012, Jennings et al. conducted a cross-sectional study, using collected data of 1898 women from the TwinsUK registry for the assessment of their flavonoid intake and vascular health. The flavonoid intake was measured through a food frequency questionnaire and the USDA database. The vascular health was measured through peripheral and central blood pressure measurements with a subset population (n = 728), also measuring arterial stiffness thought pulse wave velocity (PWV). The conclusions of this article showed that increased anthocyanin intake was associated with decreased central SBP and MAP by 3.04 ± 1.41 mmHg and 2.31 ± 1.16 mmHg, respectively, as well as decreased arterial stiffness by 0.4 ± 0.22 m/s in the subset population [27].

In a study by SS Hassellund et al., 27 male mildly-hypertensive participants were selected in order to carry out a double-blind randomized placebo-controlled crossover study. The participants were subsequently divided into two groups, placebo vs. anthocyanin. During the treatment intervals, the participants took four 80 mg capsules of anthocyanin extract twice a day for four-weeks, followed by a four-week washout. The participants had four visits during each interval, where BP measurements, lab tests, stress tests, and 24-h ambulatory BP were assessed. Although a reduction in resting supine BP was measured (reduction of 6 mmHg after approximately. 45 min of resting *p* < 0.007), this study also showed that no significant changes in sitting BP nor in the 24 h ambulatory BP were found, thus, concluding that anthocyanins could not be recommended as antihypertensive treatment in this particular population [28]. A summary of the studies can be found in Table 1.

### 3.2. Lycopene

Lycopene is a naturally occurring lipid-soluble pigment found in some plants and vegetables like tomatoes, watermelons and papaya [29]. Although it lacks provitamin A qualities, when compared to other carotenoids, lycopene is the best singlet oxygen quencher, making it a powerful antioxidant [30,31]. Several studies have been conducted regarding the anti-inflammatory properties of lycopene. In 2008, Hung et al. demonstrated the inhibitory effect of lycopene on tumor necrosis factor alpha (TNF-α) induced by the nuclear factor kappa-light-chain enhancer of the signaling pathway of activated B cells (NF-κB), and Armoza et al. achieved similar conclusion in 2012. This suggests the beneficial properties against atherosclerosis through decrease cellular endothelial adhesion [32,33].

For the above-mentioned qualities, studies have been conducted in order to test further cardiovascular properties. In 2019, 61 individuals (44 male and 17 female) were recruited for a double-blind, randomized, placebo-controlled study that assessed the blood pressure reduction qualities of lycopene in a dose dependent manner. The individuals were between 35–60 years old and were diagnosed with hypertension stage 1 or 2, without receiving any pharmacological treatment. The individuals were divided into 5 groups, in which different doses were tested: 5 mg, 15 mg and 30 mg of tomato nutrient complex (TNC) and 15 mg of synthetic lycopene and placebo, respectively. The duration of the treatment was 8 weeks with examinations every 2 weeks, which included BP measurements, pulse and physical examination. The 15 mg and 30 mg groups showed significant reduction in SBP at the end of the 8-week period, displaying the following changes: 127.2 ± 6.3 mmHg compared to 137.4 ± 5.6 mmHg at baseline and 130.0 ± 13.0 mmHg compared to 136.4 ± 7.8 mmHg at baseline, respectively. After these findings, 31 subjects agreed to continue for a four-month single-blind treatment with 15mg lycopene. The results were similar with the previous findings, SBP was reduced during the entire treatment period with results showing a decrease of 4.7 ± 6.7 mmHg compared to baseline (*p* – 0.0012) [34]. This strongly suggests blood pressure-reducing qualities of lycopene at 15 mg.

In 2005, Engelhard et al. conducted a 16-week single-blind placebo-controlled trial with 31 participants in order to evaluate tomato extract supplementation effects on blood pressure and cardiovascular risk biomarkers. All the participants had grade-1 hypertension with no other comorbidities, nor were they on any hypertensive treatment. The study was divided into a 4-week placebo period, followed by an 8-week treatment period, followed by a 4-week placebo period. During the treatment period, subjects consumed a capsule of 250 mg tomato extract containing 15 mg of lycopene. Follow-up visits were every 2 weeks. Significant blood pressure reduction was noticed during the treatment phase, with the SBP showing considerable reductions (134.02 ± 10.83 mmHg) compared to baseline (144 ± 5.99 mmHg (*p* < 0.0001)) and DBP similarly showing reductions (83.38 ± 6.6 mmHg) compared to baseline (87.44 ± 6.8 mmHg ( *p* = 0.003)) [35]. Furthermore, similar findings were also observed in 2008 during a double-blind crossover trial that compared the effects of tomato extract and placebo on patients treated for uncontrolled moderate hypertension. Two identical capsules were used, with the first containing 250 mg tomato extract (15 mg lycopene) and the other being a placebo capsule containing soya oil. Each treatment period was 6 weeks with follow-ups at baseline and every 3 weeks. The results of this study showed significant changes in SBP compared to baseline in both groups—145 ± 8.7 at baseline changing to 132.2 ± 8.6 mmHg (*p* < 0.001) and 140.4 ± 13.3 at baseline changing to 128.7 ± 10.4 (*p* < 0.001) during the supplementation with tomato extract [36]. 

Nevertheless, in 2012, Thies et al. observed no significant changes of CVD biomarkers as well as no significant changes in SBP, DBP and PWV. The study enrolled 225 moderately overweight participants (94 men and 131 women), without concomitant diseases between 40 and 65 years of age for a 16-week single-blind, randomized controlled trial. The participants began with a 4-week controlled diet low in tomato and were later divided into three intervention groups; a control group with a low tomato-based diet (<70 mg/week), a lycopene supplemented group (capsule 10 mg/day) and a high tomato-based diet (>70 mg/week). Blood pressure and PWV were measured at baseline and after intervention, whereas blood samples were taken at baseline, after 6 weeks and after 12 weeks [37]. Although the results of this study were not similar with the previous findings described in this review (Table 2), it is important to keep in mind that the populations studied were different, with the first three studies having a hypertensive population and the latter a nonhypertensive population. This may suggests that lycopene has better effects on hypertensive patients.

### 3.3. Docosahexaenoic Acid (DHA)

Docosahexaenoic acid (DHA) together with eicosapentaenoic acid (EPA) is a type of polyunsaturated fatty acids (PUFAs) belonging to the omega3 fatty acids, commonly found in oily fish. These PUFAs have been the topic of several studies. Even though guidelines from the American Heart association (AHA) and the European society of cardiology (ESC) recommend the intake of oily fish in their diet to reduce cardiovascular risk, studies have been inconclusive regarding the actual beneficial cardiovascular effects of fish oils. A recent meta-analysis strongly suggests their association with a lower risk of myocardial infarction (MI), total coronary heart disease (CHD), total cardiovascular disease (CVD) and decrease in deaths of those causes [38]. This opens the question of what other benefits fish oils might have in our health.

In 2007, Theobald et al. explored the benefits of fish oil on vascular function in 40 healthy adults between 40 and 65 years. In this crossover study, participants were treated for two periods of 3 months with a wash-out period between them lasting 4 months. The first group took three capsules/day of 500 mg of refined triacylglycerol, providing approximately 0.7 g/DHA, and the other took olive oils capsules (placebo). The results showed a decrease in DBP by 3.3 mmHg (69.1 ± 7.0 post treatment, compared to 71.3 ± 8.4 at baseline) (*p* < 0.01) [39]. Similar findings were achieved by Liu et al., who in 2011, utilized data from the Adult Health and Behavioral project in order to examine effects of omega-3 fatty acid intake on blood pressure. The study composed of 265 participants between the ages of 30 and 54 showed findings suggesting an inverse relationship between serum DHA and diastolic blood pressure. Increased serum DHA decrease clinical DBP by 2.1 mmHg and ambulatory DBP by 2.3 mmHg [40]. 

Results from a research carried out by Sagara et al. found a significant correlation in blood pressure and lipid reduction on subjects treated with DHA. The study analyzed the data from 38 middle-aged males, who were divided into two groups and underwent a 5-week treatment with 2 g of DHA/day or 1 g of olive oil (placebo). None of the subjects had any lipid or hypertensive treatment. Blood pressure and blood samples were taken at baseline and post treatment, at the beginning of the sixth week. The DHA treated group showed a decrease in SBP of 133.0 ± 12.9 compared to 141.4 ± 13.5 at baseline, and a decrease in DBP of 81.9 ± 9.3 compared to 86.7 ± 10.4 at baseline. A high-density lipoprotein cholesterol (HDL-c) increase of 16.7% (8.6 mg/dL) was also reported, and the placebo group also showed an increase in HDL-c of 9.9% (4.5 mg/dL) [41].

Furthermore, in 2019, Cheng et al. used data gathered by the National Health and Nutrition Examination Survey (NHANES) to conduct a cross-sectional study regarding the dietary intake association of omega-3 and omega-6 fatty acids and the incidence of hypertension. The gathered data covered the periods of 2007 and 2014 with a population of 18,434 participants, all being over 18 years of age. The results of this study suggests an association between increased omega-3 and omega-6 fatty acids and a decrease in incidence of hypertension [42].

These studies suggest a positive effect of omega-3 fatty acids in blood pressure reduction (Table 3). Although the exact mechanism in which omega-3 fatty acids decrease blood pressure is largely unknown, some mechanisms are suggested. Fischer et al. suggest that PUFAs inhibit the angiotensin II pathway mechanism [43]. Nestel et al. found that PUFAs increased systemic arterial compliance [44]. Van den Elsen et al. found that long-chain omega-3 PUFAs improved endothelial function in spontaneous hypertensive rats through disruption of the formation of thromboxane A₂ (TXA₂), a decrease in IL-10 and enhanced RANTES (regulated on activation, normal T-cell expressed and secreted) [45].

### 3.4. Caffeine

Coffee is a widely popular warm beverage consumed worldwide. Coffee may be one of the world’s most loved beverages. People all over the world consume almost 9 billion kg every year [46].

In addition to the stimulant effect of caffeine on the central nervous system, it has also been demonstrated to have a pharmacological effect on the cardiovascular system [47]. However, the mechanism of the action as to how it increases blood pressure is not known.

A randomized, double-blind, placebo-controlled clinical trial was conducted in 2016 on 104 young normotensive adult participants. Intervention and placebo were single drinks of coffee, caffeinated (80 mg of caffeine) or decaffeinated, respectively. The amount was 250ml per cup. The results showed a mean difference in SBP and DBP of +2.77 mmHg (*p* = 0.05) and +2.11 mmHg (*p* = 0.64), respectively. The study showed no significant rise in systolic and diastolic blood pressure on young normotensive participants after one cup of coffee [48].

A cohort study was done in 2016 by Grosso et al. which had 2725 participants who were from the Polish population of the HAIPEE project (Health, Alcohol, and Psychosocial factors in Eastern Europe) and were normotensive at the start of the study. The participants were followed up for an average of 5 years. Coffee consumption was categorized according to a standard cup of coffee: 150 mL per cup. They did not take into consideration of the dosage in milligrams of caffeine per cup. The study showed the risk of hypertension was lower for individuals having 3–4 cups per day in both sexes of non-smokers [49].

A pilot study in 2018 on 24 women was conducted where green coffee was given which contained 6mg of caffeine. The change in blood pressure values of the women following the consumption of green coffee was not statistically significant (*p* > 0.05). The pilot study did not compare effects of a placebo or other sources of caffeine. However, the metabolic changes were compared between baseline/before and after ingestion when evaluating the effects of green coffee [50].

In 2010, a significant increase in blood pressure was observed in a randomized, 2-week crossover design study involving 165 healthy men and women. It involved 6 days of dosing caffeine 80 mg × 3 daily followed by testing in a laboratory with a challenge dose of 250 mg. The other week was tested with placebos [51].

A blind and random approach study was performed in 2017. Ten healthy men in their twenties volunteered. Coffee and decaffeinated coffee (Placebo) were compared. After a 10-min baseline measurement, the participant would drink either coffee or the placebo. The study showed that mean arterial pressure and systolic blood pressure were higher at times of 30 and 60 min after drinking the coffee when compared to the placebo [52].

Table 4 presents a summary of reviewed studies showing conflicting evidence regarding blood pressure changes caused by caffeine. Therefore, a consensus cannot be established whether caffeine has an effect of increasing or reducing blood pressure. More studies need to be analyzed and more data needs to be gathered in order to conclude whether caffeine has an effect.

### 3.5. Dietary Fiber

Dietary fiber is a type of carbohydrate mostly found in plants and can be classified into soluble/fermentable (pectin, gums, mucilages) and insoluble/non- or partially fermentable (cellulose, hemicellulose, lignin) [53]. Dietary fiber has gained increased relevance for the past years, due to several positive health effects, including reduced risk of colorectal cancer, metabolic syndrome, diabetes, cardiovascular disease and death [54,55,56,57,58]. Moreover, it has also been associated with reduced weight, waist circumference and body fat percentage [59,60]. Dietary fiber also helps to reduce cholesterol uptake by binding it to the gut [61,62].

In a meta-analysis by Clark CCT et al., significant data was found on the effect of dietary fiber on hypertensive patients in randomized controlled trials. A total of 11 trials with 592 participants were analyzed. It revealed a reduction of 2.04 mmHg in SBP (95% confidence interval, *p* < 0.001). The analysis also revealed that the effect of psyllium was stronger in participants with a higher baseline blood pressure [63].

In 2018, Machoene D. Sekgala et al. provided a study with 627 young adults in villages in Limpopo, Africa. Dietary fiber intake was measured by a validated 24-h recall method. The majority of participants had reported an extremely low intake of fiber, barely hitting 2 g per day. Log total fiber was inversely proportional with SBP (*p* = 0.051) [64].

A systematic review and meta-analysis of randomized controlled trials by K. Khan et al. found that viscous soluble fiber had a reducing effect on both systolic and diastolic blood pressure. Diets rich in beta-glucans, with median difference of 4 g between the placebo and control group showed a reduction of 2–9 mmHg for SBP and 1–5 mmHg for DBP (95% CI). It was observed that the effects could be stronger in individuals who were originally hypertensive [65].

In 2005, a meta-analysis of randomized placebo-controlled trials of dietary fiber intake and blood pressure was completed by Streppel et al., where a total of 1404 subjects were included, and sample size ranged from 12 to 201 subjects. In 24 randomized controlled trials, fiber intake was at a mean dose of 11.5 g/dL. It caused *a* non-significant change to SBP (−1.13 mmHg) but a significant reduction in DBP (−1.26 mmHg). The effect was stronger on participants aged above 40 years, but this was only significant for SBP. Changes in blood pressure became less pronounced with increased dose of fiber. Blood pressure reductions tended to be larger in hypertensive populations [66].

Becerril-Alarcon et al. explored the effects of inulin supplementation on 38 middle aged women with breast cancer undergoing neoadjuvant chemotherapy. A total of 19 of the participants had an SBP of > 130 mmHg before the supplementation. The participants were given 15 g of powdered agave inulin or 15 g of maltodextrin as a placebo. The powders were dissolved in 250 mL of water, and were taken during breakfast. The supplementation lasted for 21 days at the same time as they underwent chemotherapy with doxorubicin and cyclophosphamide for the first time. The study found that inulin supplementation had a significant reduction in SBP of 4.21 mmHg (*p* < 0.001) and no significant changes regarding DBP [67].

Table 5 lists a comparison of studies used regarding this topic. Dietary fiber has several beneficial effects that are relevant for reducing the risk of cardiovascular disease such as; reduced weight, reduced risk of metabolic syndrome and diabetes, reduced waist circumference, body fat percentage and cholesterol. It is also important to appreciate the effect of reducing the risk of colorectal cancer. There seems to be a large amount of studies providing similar results and it could be argued that fiber undoubtedly has an effect of reducing blood pressure.

### 3.6. Licorice

Licorice is a herb and is the root of Glycyrrhiza glabra from where you can extract sweet flavor. The plant is native to the Middle East, southern Europe and parts of Asia. It has been used as traditional medicine for the treatment of peptic ulcer, hepatitis C and respiratory and skin diseases [68]. Several components have been isolated from licorice: triterpene saponins, flavonoids, isoflavanoids, chalcones, and most importantly, the main biologically active component: glycyrrhizic acid. Licorice flavors are used as candies or sweeteners in beverages. Licorice is associated with increased blood pressure and excessive consumption can cause a clinical picture similar to primary hyperaldosteronism [69]. Licorice induces its effect on blood pressure primarily by the inhibition of renal 11-β-hydrogenase type II enzyme by 3 β-monoglucuronyl-18 β-glycyrrhetinic acid and 18 β-glycyrrhetinic acid. Water and sodium retention in the kidney increases blood volume and elevates blood pressure [70].

Additionally, Gomez-Sanchez E.P. et al. found that infusion of 18 β-glycyrrhetinic acid into rat brain resulted in an elevation of blood pressure without affecting renal sodium and water resorption [71].

Leskinen et al. concluded that only 290–370 mg licorice taken daily resulted in an elevated systolic blood pressure and elevated diastolic blood pressure after only two weeks [72]. Sigurjónsdóttir et al. also found that a daily dose of only 50 g of licorice was sufficient to cause a noticeable increase in systolic blood pressure within a period of two weeks [73]. Similar observations were also reported by Penninkilampi R. et al. in a meta-analysis: 18 studies were analyzed (337 patients) and SBP and DBP seemed to rise depending on the dose, suggesting a public recommendation of avoiding excessive licorice intake [74].

A 12-week experiment was done by van Gelderen et al. with 39 healthy female volunteers, and they proposed a no-effect level of 2 mg/kg 18 β-glycyrrhetinic acid per day (equivalent to 6 g licorice for a person of 60 kg bodyweight [75].

In 1994, it was observed by Basso A. et al. that a 63-year old type 2 diabetes mellitus patient was treated for orthostatic hypotension using licorice (3 g of 18 β-glycyrrhetinic acid/day) as treatment. The patient’s blood pressure increased from 90/60 mmHg to 130/80 mmHg in an upright position in 7 days of therapy. Therefore, there might be some indications that licorice can be suggested to be used in clinical therapy, but this must be further investigated in a double-blind, randomized, controlled trial to avoid bias [76].

From the abovementioned studies (Table 6), there seem to be overwhelming evidence showing that licorice intake causes an increase in blood pressure. There needs to be expressed caution about licorice intake due to its popularity and increased number of cardiovascular events in recent years. In addition, licorice-induced hypokalemia needs to be taken seriously. However, it needs to be established whether licorice can be used to treat hypotension and whether it is suitable to be recommended to patients, because a treatment option with licorice must be monitored by the healthcare provider or by home measurement in order to control blood pressure.

### 3.7. Epigallocatechin-3-gallate (EGCG)

Epigallocatechin-3-gallate (EGCG), is a major catechin found in green tea (*Camellia sinensis*) with known antioxidant and anti-inflammatory properties [77,78,79,80]. EGCG has been the source of numerous research papers for the past years claiming different health benefits, such as an antiproliferative effect on human colorectal cancer cells, as well as promoting cardiovascular and metabolic health [81,82]. Yang et al. noted that green tea consumption decreased hypertension risk in a selected Chinese population, thus opening further questions regarding green tea compounds and their effect on human health [83].

In a randomized study conducted by Brown et al., 100 nondiabetic, nonsmoker, obese or overweight male subjects were enrolled to assess the effectiveness of epiallocatechin-3-gallate on insulin resistance as well as associated metabolic risk factors. The subjects were randomly assigned into a placebo group, taking 400 mg lactose capsules or into an EGCG group taking 400 mg EGCG capsules. The capsules were taken twice daily for an 8-week period. The results of this study showed no significant changes in insulin resistance, total cholesterol, HDL-cholesterol and LDL-cholesterol, but they did show a positive DBP reduction (mean change; placebo –0.058 mmHg; EGCG –2.68 mmHg (*p* = 0.014)) [84].

Results from a randomized study, carried out by Nantz et al. showed that a standardized twice-a-day dose of decaffeinated green tea products (containing L-theanine and EGCG) effectively reduced SBP and DBP at 3 weeks and only SBP after 3 months. Hundred and eleven healthy men and women between 21–70 years old volunteered for this study and were randomly assigned to either the placebo (PBO) group or the *Camellia sinensis* compounds (CSC) group, with follow-ups at baseline, after 3 weeks and after 3 months. There results were as follows: SBP was 130 ± 6.88 mmHg at baseline, 126 ± 6.3 mmHg at 3 weeks and128 ± 6.3 mmHg at 3-months, while DBP was 80.2 ± 4.15 mmHg at baseline, 76 ± 4.2 mmHg at 3 weeks, and 79 ± 4.2 mmHg at 3 months [85].

In 2012, Bogdanski et al. conducted a 3-month, double-blind, placebo-controlled trial with 56 obese, hypertensive males and females for the purpose of assessing the effect of green tea extract on insulin resistance and associated cardiovascular risk factors. The subjects were randomly assigned to either one capsule/day of green tea extract (GTE) (containing 208 mg EGCG) or placebo (containing cellulose) with results showing a decrease in SBP and DBP (145 ± 10 mmHg at baseline compared to 141 ± 8 mmHg after 3 months and 88 ± 4 mmHg at baseline compared to 84 ± 3 mmHg after 3 months, respectively) as well as a considerable reduction in LDL-C, TG, insulin resistance and an increase in HDL-C [86].

In 2018, 120 healthy subjects between the ages of 20 and 60 were subjects of a 12-week, parallel study conducted by Maeda-Yamamoto et al., whose aim was to determine if visual function and BP could be improved by green tea containing anthocyanin. The subjects were divided into three groups: the first group consumed barley extract (without catechins), the second group consumed “Sunrouge” (containing 323.6 mg EGCG and 11.2 mg anthocyanin) and the third group consumed “Yubukita” (containing 322.2 mg EGCG). The results of this study showed no significant BP or visual improvement in the “Yubukita” group. Moreover, the “Sunrouge” group showed an increase in blood pressure (SBP of 123.3 ± 12.9 mmHg at baseline, compared to 129.8 ± 12.7 mmHg after 12 weeks, *p* < 0.01 and DBP of 77.1 ± 8.5 mmHg at baseline, compared to 82.4 ± 8.2 mmHg after 12 weeks, *p* < 0.01) at bedtime, as well as improvement of the eye accommodation ability in subjects below the age of 45 [87].

The mechanism by which EGCG reduces blood pressure is not fully understood to date, and several mechanism have been suggested. Tea polyphenols have been shown to have an inhibitory effect on renin activity [88]. Persson et al. suggest that green tea extracts inhibit angiotensin-converting-enzyme (ACE) activity as well as increasing NO productions [89]. Antonello et al. found that the antihypertensive effects of GTE were likely due to their scavenging of superoxide anion generation [90]. Lorenz et al. suggest that increased eNOs activity increases vasorelaxation [91]. This finding was also indicated by Kim et al., however, a different pathway was suggested [92]. These findings may imply that several mechanisms may be involved in the BP regulation of EGCG.

Moreover, it is important consider that the dosages of the articles presented in this review (Table 7) could be equivalent to over ten cups of green tea.

### 3.8. Bioactive Peptides

Protein that is found in foods functions as nutrients, but proteins can also provide beneficial physiochemical roles that promote health. For example, in vitro and in vivo studies have shown the possible role of cancer prevention (specifically apoptosis in human leukemic cells) of bioactive peptides [93]. Increased consumer awareness of the effects of foods has led to a strong drive for research and production of bioactive peptides in foods. The most studied food derived antihypertensive peptides are IPP (Ile-Pro-Pro) and VPP (Val-Pro-Pro) [94]. 

Bioactive peptides can be released during enzymatic proteolysis and fermentation. Many low molecular weight peptides have great ACE inhibition because low molecular weight peptides have a high rate of absorbency in the body. The ACE inhibitory effect is related to the degree of enzymatic hydrolysis and composition of the sequence of the peptide in question. When compared with synthetic ACE inhibitor drugs, ACE inhibitors derived from natural food have minimal side effects like cough, increased blood potassium levels, fatigue and fetal disorder. They have been mainly discovered by hydrolyzing proteins with food-grade proteolytic enzymes to release several peptide fragments into the hydrolysate. Proteins can also be fermented by bacteria through the bacteria’s proteolytic enzymes. The bioactive peptides in the hydrolysate can then be used as a functioning food and purified into nutraceuticals for nonpharmacological therapy. Bioactive peptides can be found in nature in plants, animals, fungi and microbes [95].

A comprehensive meta-analysis was published in 2015 containing 30 studies involving 33 trials with a total of 2200 participants (who were randomized) that were published between 1996 and 2014. They tested the effect of lactotripeptides (LTP) on blood pressure. It showed LTP consumption reduced office SBP by 2.95 mmHg (*p* < 0.001) and reduced office DBP by 1.51 mmHg (*p* < 0.001). The intervention duration varied between 4–21 weeks. Blood pressure reduction was more evident in the Japanese and Finnish studies [96].

In 2016 the “Whey2Go” study was published. It was a randomized controlled trial of 38 participants who were given 2 × 28 g whey protein/day, 2 × 28 g caseinate/day, or 2 × 27 g maltodextrin/day (control). They had a 4-week wash-out. The results indicated significant reduction in both SBP (–3.9 mmHg) and DBP (–2.5 mmHg). *p* = 0.050 for both [97].

A randomized controlled trial was published in 2005 that studied the effect of soybean protein on blood pressure. 302 participants from 35 to 64 years of age were included in the study. Participants were given randomly 40 g of isolated soybean protein supplements per day or complex carbohydrate (control) for 12 weeks. The results showed a significant reduction in SBP (–4.31 mmHg) and DBP (–2.76 mmHg). *p* < 0.001 for both values. The study did not examine whether the reduction in blood pressure was due to the protein or the isoflavones in soybean [98].

In 2001 a study about the effects of “katsuobushi” consumption and blood pressure was published. Katsuboshi is dried, fermented and smoked skipjack tuna (*Katsuwonus pelamis*). It is a traditional Japanese food which is known to inhibit the angiotensin I-converting enzyme (ACE). From this, they used hydrolyzate “katsuobushi oligopeptide” (KO) and a stronger (S-KO) by ultrafiltration for the consumption for participants. A total of 65 participants with borderline and mildly hypertensive participated in the study. The participants were monitored for 10 weeks. Ingestion of a placebo and SKO tablets (1.5 g/day) were compared. S-type KO significantly lowered blood pressure at a dose that can be used as a daily dietary supplement [99].

Table 8 shows a summary of the above reviewed article.

## 4. Discussion

In this review, several bioactive compounds and their effects on blood pressure were analyzed. Although not conclusive, there is good evidence suggesting the blood pressure-reducing effect of lycopene, DHA, and fiber, but further studies should be conducted in order to find optimal dosages and populations. There is also some evidence suggesting similar qualities in anthocyanin, but to a lesser degree. More evidence is needed in order to be able to make any definitive conclusions in addition to finding optimal dosages and type of population. There is good evidence regarding *EGCG*, but larger and longer studies are needed before any definitive conclusions should be made. It is also of great importance to assess optimal dosages and populations for the maximization of its effects.

Regarding bioactive peptides, the evidence shown in this review suggests a positive effect on blood pressure reduction, but larger quantitative studies need to be performed in order to draw a conclusive correlation. Nevertheless, the evidence to date is promising. There is limited available data regarding caffeine, which shows inconclusive and even conflictive evidence, thus, more studies are needed before any assumption can be made. As for licorice, there is substantial evidence showing its blood pressure-increasing effects and patients should be advised to be cautious and discouraged from its consumption. 

Apart from licorice, all other substances have been suggested, to a certain degree, to have a positive effect on the cardiovascular system. Thus, their consumption should still be advised in populations at risk. 

## Figures and Tables

**Table 1:** Comparison of studies.

Author and Reference Number	Number of Participants	Study Design	Participants Description	Duration	Findings
Cassidy et al., 2011 [26]	156,952	Prospective	Healthy women and men	14 years	Risk of hypertension
Jennins et al., 2012 [27]	1898	Cross-sectional	Hypertensive women	–	cSBP Map Arterial stiffness
Hasselund et al., 2011 [28]	27	Crossover	Mildly hypertensive men	8 Weeks	Slight of resting BP No other significant changes

**Table 2:** Comparison of studies.

Author and Reference Number	Number of Participants	Study Design	Participants Description	Duration	Findings
Wolak et al., 2019 [34]	61	Double-blind	Hypertensive women and men	8 weeks	SBP
Wolak et al., 2019 [34]	31	Single-blind	Hypertensive women and men	4 months	SBP
Engelhard et al., 2005 [35]	31	Double-blind	Grade-1 hypertension women and men	16 weeks	SBP and DBP
Paran et al., 2008 [36]	50	Crossover	Moderate hypertensive women and men	12 weeks	SBP
Thies et al., 2012 [37]	225	Single-blind	Moderate overweight women and men	16 weeks	No significant changes

**Table 3:** Comparison of studies.

Author and Reference Number	Number of Participants	Study Design	Participants Description	Duration	Findings
Theobald et al., 2007 [39]	40	crossover	Healthy women and men	10 months	DBP
Liu et al., 2011 [40]	265	Observational study	Healthy women and men	N/A	Resting DBP 24-h DBP
Sagara et al., 2011 [41]	38	Double-blind	Hypertensive and/or hypercholesterolemic men	5 weeks	SBP DBP HDL-c
Cheng et al., 2019 [42]	18 434	Cross-sectional	General population	7 years	Risk of hypertension

**Table 4:** Comparison of studies.

Author and Reference Number	Number of Participants	Study Design	Participants Description	Duration	Findings
Teng CL et al., 2016 [48]	104	Double-blind	Normotensive young adults women and men	-	No significant changes
Grosso G et al., 2016 [49]	2725	Prospective	Normotensive women and men	Average of 5 years	Risk of hypertension
Acar-tek et al., 2018 [50]	24	Pilot	Healthy women		No significant changes
Farag et al., 2010 [51]	165	Crossover	Healthy women and men	2 weeks	↑BP
Washio et al., 2017 [52]	10	Randomized and blinded	Healthy men	-	MAP

**Table 5:** Comparison of studies.

Author and Reference Number	Number of Participants	Study Design	Participants Description	Duration	Findings
Clark CCT et al., 2020 [63]	592	Meta-analysis	Hypertensive women and men	-	SBP
Sekgala et al., 2018 [64]	627	Cross-sectional	Random	20 years	BP
Khan et al., 2018 [65]	2773	Meta-analysis of RCT	-	≥ 4 weeks	SBP DBP
Streppel et. al., 2005 [66]	1404	Meta-analysis of RCT	-	-	DBP
Barcerril-Alarcón et al., 2019 [67]	38	Double-blind	Normotensive and mildly hypertensive women with breast cancer	21 days	SBP

**Table 6:** Comparison of studies.

Author and Reference Number	Number of Participants	Study Design	Participants Description	Duration	Findings
Leskinen et al., 2014 [72]	20	Open-label	Normotensive women and men	2 weeks	SBP DBP
Sigurjónsdóttir et al., 2001 [73]	64	Intervention	Normotensive women and men	2–4 weeks	SBP
Penninkilampi R. et al., 2017 [74]	337	Meta-analysis	Healthy women and men	1–8 weeks	SBP DBP
van Gelderen et al., 2000 [75]	39	Comparative	Healthy women	12 weeks	No changes

**Table 7:** Comparison of studies.

Author and Reference Number	Number of Participants	Study Design	Participants Description	Duration	Findings
Brown et al., 2008 [84]	100	Parallel	Obese or overweight men	8 weeks	DBP
Nantz et al., 2008 [85]	111	Parallel	Healthy men and women	3 months	SBP DBP
Bogdanski et al., 20012 [86]	56	Parallel	Obese, hypertensive men and women	3 months	SBP DBP LDL-C TG insulin resistance HDL-C
Maeda-Yamamoto et al., 2018 [87]	120	Parallel	Healthy men and women	12 weeks	DBP

**Table 8:** Comparison of studies.

Author and Reference Number	Number of Participants	Study Design	Participants Description	Duration	Findings
Frekete et al., 2015 [4]	2200	Meta-analysis	Normotensive and hypertensive Men and women	4–21 weeks	BP
Frekete et al., 2016 [5]	38	Double-blind	Mild hypertensive Male and females	8 weeks	BP
He et al., 2005 [6]	302	Double-blind	Hypertensive Men and women	12 weeks	BP
Fujita et al., 2001 [7]	65	Double-blind	Males and Females Hypertensive	10 weeks	BP
